# Increased feelings of external influence during instructed imaginations in patients with psychotic disorder

**DOI:** 10.1038/s41598-025-10439-7

**Published:** 2025-07-22

**Authors:** Kathrin N. Eckstein, David Rosenbaum, Anna Camera, Lisa Röhrig, Matthias L. Herrmann, Dirk Wildgruber

**Affiliations:** 1 Department of Psychiatry and Psychotherapy, Tübingen Center for Mental Health (TüCMH), University of Tübingen, Tübingen, Germany; 2 Clinic for General Psychiatry and Psychotherapy, Zentrum für Psychiatrie (ZfP), Klinikum am Weissenhof, Weinsberg, Germany; 3 Translational Research Office, Medical Science Division, University of Oxford, Oxford, UK; 4 Division of Neuropsychology, Center of Neurology, Hertie-Institute for Clinical Brain Research, University of Tübingen, Tübingen, Germany; 5 Department of Neurology and Neuroscience, Medical Center, University of Freiburg, Freiburg, Germany

**Keywords:** Delusion of influence, Emotional Valence, Response latency, Schizophrenia, tDCS, Hand touch, Neuroscience, Physiology, Psychology, Diseases, Medical research, Pathogenesis, Signs and symptoms

## Abstract

Feelings of external influence are a hallmark of psychotic disorders, but mitigated forms can also be observed in healthy persons. A newly developed paradigm designed to induce feelings of external influence through verbal information (influence will be attempted/not-attempted) and physical setup (presence of tDCS-device or hand touch) was carried out in a pilot study in 21 patients with psychosis and in 22 healthy controls. A higher prevalence of induced feelings of external influence was observed in patients with psychosis. Furthermore, in healthy controls the impact of verbal information outweighed the impact of physical setup, whereas in patients the setup had a stronger influence. Moreover, the intensity of induced feelings of external influence correlated with measures of psychotic symptom severity (PANSS positive score, SAPS, AMDP ego-disorder) as well as response latencies during the estimation process. These findings indicate that feelings of external influence can be reliably induced in healthy controls and in patients with psychotic disorders. Stronger effects of physical setup in patients might be related to disorder-specific biases regarding the impact of initial perceptual impressions of features of the physical environment on belief formation, whereas the attenuated response to explicit verbal information about attempted influence might reflect reduced trust in other people’s statements.

**Supplementary Information:**

The online version contains supplementary material available at 10.1038/s41598-025-10439-7.

## Introduction

Psychotic disorders are observed worldwide with a life-time prevalence of 0.7%^1,2^ and have strongly burdening effects^2,3^. In a narrow definition, psychotic symptoms comprise hallucinations (e.g. hallucinatory voices) and delusions (e.g. false beliefs of being persecuted, observed or controlled by others). Patients with psychotic disorders, however, often additionally show impairments in basic cognitive functions, such as working memory or attention^4^, disorganized thinking, abnormal motor behaviour (e.g. catatonia) and negative symptoms (e.g. avolition, anhedonia, asociality, affective flattening and alogia)^5^. Psychotic disorders include schizophrenia, schizotypal personality disorders, schizoaffective disorder, psychosis due to other conditions such as substance abuse, and psychosis in the context of other mental disorders such as depression or bipolar disorder^5^. Treatment options for psychotic disorders include antipsychotic medication as a first line treatment and additional psychotherapy. However, up to date prognosis for the course of the disease is in most cases not very encouraging^6^. Research on the core symptoms of psychotic disorders might improve our understanding of those diseases and give guidance towards new treatment approaches. Regarding the key symptoms, delusions of influence (i.e. the false belief that other people or external agents are covertly exerting powers over oneself) are a hallmark of psychotic disease and are a predictor of imminent disease onset and severity^7–10^. Although psychosis is often seen as a binary construct, attenuated forms of external influence presentation can also be found in healthy individuals^11^. This points towards a rather continuous spectrum of experience rather than a categorical presence vs. absence of symptoms.

**Fig. 1:**
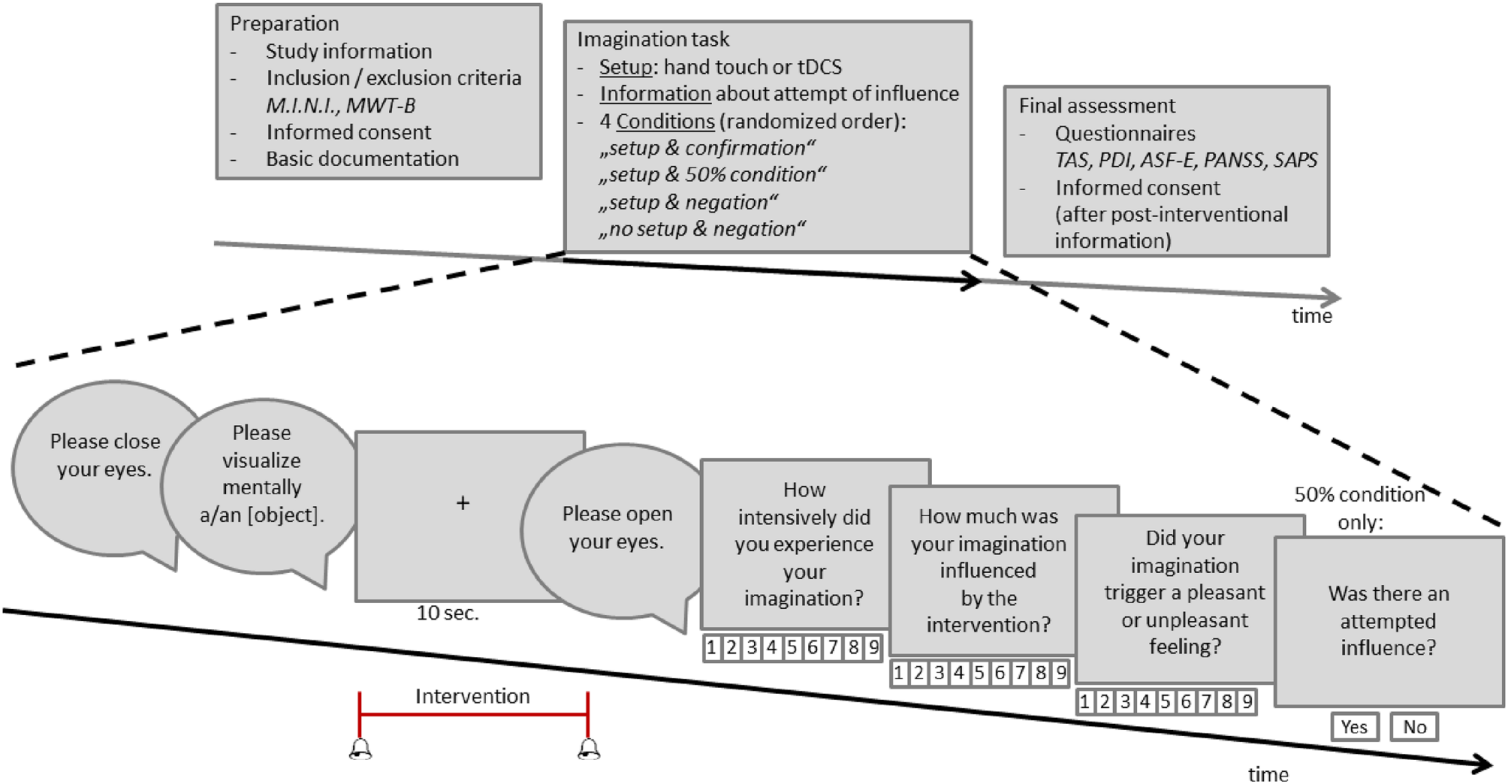
Experimental design. Upper row: Following study preparation, the physical setup was prepared (hand touch or tDCS in two separate sessions) and participants were informed whether external influence was attempted in the next trial block (confirmation / negation / 50% condition). Following the completion of the imagination task, the final assessments were carried out (including a post-interventional informed consent). Lower row: The imagination task started with the spoken request to close one’s eyes and mentally visualize an object for 10 seconds. The beginning and the end of the imagination period were signalled by a short sound (%). During this imagination the intervention was carried out according to the respective, randomized condition comprising setup (hand touch or tDCS) and information on whether external influence was attempted (confirmation / negation / 50% condition). After the imagination period the participants were asked to open their eyes again (all oral instructions are pictured in speech bubbles). Participants were asked to answer questions regarding the intensity of their imagination, the extent of perceived external influence and the emotional valence of their imagination on a 9-point Likert scale ranging from 1 = very low/unpleasant to 9 = very high/pleasant on the computer keyboard in front of them. In the 50% condition an additional question was asked concerning the assumption of whether external influence was attempted in the last trial (yes/no) (All instructions presented on the screen are displayed in rectangular boxes).

We wanted to further substantiate this concept by assessing healthy individuals as well as patients with psychotic disorder with a newly developed paradigm to modulate feelings of external influence during instructed imaginations^12^. To this end, in this pilot study 21 patients and 22 healthy controls participated in an imaginary task while feelings of external influence were induced via two experimental setups: a transcranial direct current stimulation (tDCS) device and physical hand touch. During different setup conditions (tDCS/hand touch vs. no setup), subjects were confronted with different conditions regarding additional verbal information, summing up to four different experimental conditions (see Fig. 1): “setup & confirmation” (setup applied and information that an influence attempt will be made), “setup & negation” (setup applied, but negated attempt of influence), “no setup & negation” (no setup and negated attempt of influence) and “setup & 50% condition” (setup applied and information that influence attempts will be made in 50% of trials).

The main aim of the pilot study was to test the following hypothesis: Feelings of external influence during instructed imaginations are stronger in patients with psychotic disorder in comparison to healthy controls across all experimental conditions. In addition, we conducted exploratory analyses to compare the relative impact of the physical setup (tDCS/hand touch vs. no setup) with the impact of the information component (“setup & affirmation” vs. “setup & negation”) on the intensity of feelings of external influences and response times in patients and controls. Moreover, associations between psychopathological measures and feelings of external influence and response latencies were evaluated. Regarding the effectiveness of both interventions (tDCS and hand touch), we assumed the replication of previously published findings in healthy participants^12^: (1) Both interventions (consisting of the two modulating factors physical setup and information about attempted influence) modulate feelings of external influence during instructed imaginations. (2) The presence of the physical setup (tDCS device as well as hand touch) augments feelings of external influence compared to the condition without setup, even if the attempt to influence the imagery is explicitly negated. (3) The information about attempted influence increases feelings of external influence during both interventions.

## Results

### Participants’ sample description

Patients with psychotic disorder and healthy controls differed concerning the neurocognitive and psychopathological examination (crystallized intelligence (MWT-B)^13^, assessment of symptoms of ego disorders (AMDP ego disorder)^14^, stability of attributions concerning positive events (ASF-E stable positive)^15^, and proneness to delusional experience (PDI)^16^, but not in any other variable (see Table 1).

**Table 1:** Sociodemographic and psychometric characteristics of the participants.

	Patients with psychotic disorder (*n* = 21)	Healthy controls (*n* = 22)	*p*
Age (years)	36.8 ± 7.9	37.6 ± 14.6	0.610
Sex	m = 11, f = 10	m = 9, f = 13	0.547
First language	German = 18, Other = 3	German = 20, Other = 2	0.664
Educational level	High school graduation (*n* = 13), general certificate of secondary education (*n* = 3), lower secondary school graduation (*n* = 5)	High school graduation (*n* = 18), general certificate of secondary education (*n* = 3), lower secondary school graduation (*n* = 1)	0.079
Professional qualification	Academic degree (*n* = 7), apprenticeship (*n* = 5), None (*n* = 9)	Academic degree (*n* = 7), apprenticeship (*n* = 6), None (*n* = 9)	0.967
Order of sessions (first session)	tDCS = 11, hand = 10	tDCS = 10, hand = 12	0.763
MWT-B	27.0 ± 5.4	31.2 ± 3.4	**0.007**
Familial history of psychiatric disease	No = 13, Yes = 8 (3 psychotic disorder, 2 affective disorder, 3 addiction disorder)	No = 18, Yes = 4 (4 affective disorder)	0.185
AMDP ego disorder	10.4 ± 6.2	2.3 ± 3.3	**< 0.001**
ASF-E stable positive	68.0 ± 11.2	76.7 ± 12.0	**0.030**
ASF-E global positive	74.3 ± 14.2	78.7 ± 16.6	0.219
ASF-E internal positive	70.4 ± 10.4	71.5 ± 11.1	0.324
ASF-E stable negative	62.4 ± 12.9	62.5 ± 15.2	0.932
ASF-E global negative	64.0 ± 17.9	52.4 ± 21.2	0.055
ASF-E internal negative	68.8 ± 10.6	64.0 ± 10.0	0.062
PDI	170 ± 142.4	43.3 ± 34.0	**0.002**
TAS	48.1 ± 28.7	41.2 ± 20.0	0.519
PANSS positive	13.6 ± 6.5	-	-
PANSS negative	16.8 ± 7.4	-	-
PANSS general	29.3 ± 8.6	-	-
SAPS total scale	20.1 ± 23.0	-	-

tDCS = transcranial direct current stimulation; m = male; f = female; MWT-B = Mehrfachwahl-Wortschatz-Intelligenztest (Multiple-choice Vocabulary Intelligence Test); AMDP = Arbeitsgemeinschaft für Methodik und Dokumentation (Working group for methodology and documentation); ASF-E = Attributionsstilfragebogen für Erwachsene (Attribution style questionnaire for adults); PDI = Peters et al. Delusions Inventory; TAS = Tellegen Absorption Scale; PANSS = Positive and Negative Syndrome Scale; SAPS = Scale for the Assessment of Positive Symptoms. Mean values, standard deviations and p values are shown (p values < 0.05 are considered significant and printed in bold).

### Augmented feeling of external influence in patients with psychotic disorder

**Fig. 2:**
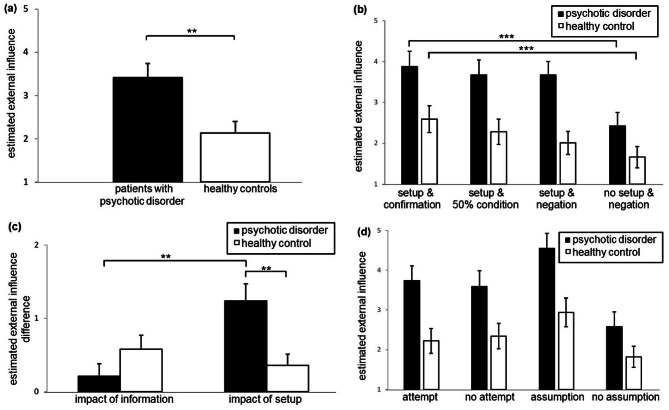
Feelings of external influence during instructed imagination in patients with psychotic disease and healthy controls. (**a**) Mean estimated external influence ratings across all conditions for patients with psychotic disease (black) and healthy controls (white). (**b**) Mean estimated external influence ratings separated for the four different conditions (“setup & confirmation”, “setup & 50% condition”, “setup & negation” and “no setup & negation”) and the two groups (patients and healthy controls). (**c**) The modulating factor “impact of information” represents the difference between the estimation of external influence in the condition “setup & confirmation” minus “setup & negation”. The modulating factor “impact of setup” is the difference of the estimated feelings of external influence between the conditions “setup & negation” and “no setup & negation”, which differ in the presence and absence of the setup, respectively. (**d**) During the “setup & 50% condition” participants were informed that an influence was attempted in half of the trials, but the participants did not know in which ones. Mean values were calculated for the trials with attempted influence and without attempted influence. In addition, the participants were asked after each trial for their assumption concerning the attempt of external influence, giving a yes or no answer. Trials were also separated concerning the answer of the participants into trials with assumed influence and not assumed influence. Patients with psychotic disorders are represented in black bars, healthy controls in white bars. Mean values and standard error of the means are depicted, ** *p* < 0.005.

We hypothesised that patients would exhibit an augmented feeling of external influence in comparison to healthy controls in all conditions and for both setups. The mean estimation of external influence across all conditions and setups was 3.41 ± 0.33 in patients with psychotic disorder and 2.13 ± 0.27 in healthy controls. Repeated measures ANOVA comprising the four conditions (“setup & confirmation”, “setup & 50% condition”, “setup & negation”, “no setup & negation”) and the two interventions (tDCS, hand touch) as within-subject factors and group (patients with psychotic disorders, healthy controls) as between-subject factor revealed a statistically significant difference between the two groups concerning their mean estimation of external influence during the instructed imaginations (F(1, 33) = 6.639, *p* = 0.015, partial ɳ_p_^2^ = 0.167). The results were corroborated by nonparametric post-hoc testing (Z = -2.928, *p* = 0.003, *r* = 0.447) (see Fig. 2a). Moreover, a significant main effect of conditions was observed (F(2.006, 66.204) = 18.478, *p* < 0.001, partial ɳ_p_^2^ = 0.359), while we did not find a significant condition-by-group interaction (F(2.006, 66.204) = 2.187, *p* = 0.120, partial ɳ_p_2 = 0.062, Fig. 2b). The two types of intervention (tDCS, hand touch) did not show significant differences. Additionally nonparametric post-hoc testing showed a significant difference between the four experimental conditions in patients (Chi-Quadrat(3) = 32.36, *p* < 0.001, *n* = 21) as well as in healthy controls (Chi-Quadrat(3) = 22.92, *p* < 0.001, *n* = 22), and in both groups a significant difference between the condition “setup & confirmation” and “no setup & negation” (both Z< -3.334, *p* < 0.001, *r* > 0.710).

### Differential impact of setup and information in patients with psychotic disorder and healthy controls

The four experimental conditions mentioned above (“setup & confirmation”, “setup & 50% condition”, “setup & negation”, “no setup & negation”) comprise variations of two different modulating factors (physical setup and information on attempted influence). To disentangle the respective effects on modulation of feelings of external influence, we calculated two further variables: The “impact of information” represents the difference of the estimated external influence between the conditions “setup & confirmation” and “setup & negation”, as these two conditions differ just in the information given to the participants. The “impact of information” was 0.21 ± 0.17 for patients and 0.58 ± 0.19 for healthy controls. Accordingly, the “impact of setup” is the difference of the estimated feelings of external influence between the conditions “setup & negation” and “no setup & negation”, which differ only in the presence and absence of the setup, respectively. The “impact of setup” was 1.24 ± 0.23 for patients and 0.36 ± 0.15 for healthy controls.

The repeated measures ANOVA comprising these two modulating factors (“impact of information”, “impact of setup”,) as the within-subject factor and group (patients with psychotic disorders, healthy controls) as the between-subject factor showed further differences between the groups, reflected by a significant interaction of modulating factor*group (F(1, 41) = 8.843, *p* = 0.005, partial ɳ_p_^2^ = 0.177). Non-parametric post-hoc testing confirmed a statistically significant difference between patients and healthy controls (Z = -3.138, *p* = 0.002, *r* = 0.479). In patients, the effect of information and setup differed significantly (Z = -2.726, *p* = 0.005, *r* = 0.595) (see Fig. 2c).

To further support our results, we calculated the difference of the estimated external influence between the modulating factors “impact of information” and “impact of setup”. This difference entails − 1.03 ± 0.32 in patients with psychotic disorder and 0.22 ± 0.23 in healthy controls, highlighting that the “physical setup” has a more pronounced effect on patients with psychotic disorder than “information”, and vice versa for healthy controls. This group difference was statistically significant (Z = -2.467, *p* = 0.013, *r* = 0.376). Only the impact of information in patients was not significantly different from zero (*p* = 0.230), all others had a significant effect (*p* ≤ 0.024) (see Fig. 2c).

### Estimated external influence during the “setup & 50% condition”

In the “setup & 50% condition” patients with psychotic disorder as well as healthy controls judged the attempt of influence correct in about half of the trials (0.51 ± 0.01 and 0.52 ± 0.01, respectively). The accuracy rate did not differ statistically significantly from chance level probability of 0.5 (*p* = 0.586 and *p* = 0.196, respectively), neither was there a significant difference between the two groups (Z = -0.253, *p* = 0.821, *r* = 0.036). Applying RM ANOVA for the “setup & 50% condition” concerning the estimated external influence with intervention as within-subject factor and group as between-subject factor, we found a significant difference between patients with psychotic disorder (3.67 ± 0.37) and healthy controls (2.28 ± 0.31) (F(1, 33) = 5.924, *p* = 0.021, partial ɳ_p_^2^ = 0.152). Post-hoc nonparametric testing corroborated the results (Z = -2.770, *p* = 0.005, *r* = 0.422). There was no significant interaction of condition*group (F(1, 33) = 2.804, *p* = 0.103, partial ɳ_p_^2^ = 0.078) (see Fig. 2d). Mean number of trials with assumed external influence was 12.10 ± 1.19 in patients with psychotic disorder and 10.07 ± 1.13 in healthy controls. There was no statistically significant difference between the two groups (Z = -1.472, *p* = 0.144, *r* = 0.224).

### Correlation of the estimated external influence with psychopathological measures of psychotic symptom severity

In all 43 participants the degree of ego-disorder symptoms was assessed using the AMDP manual^14^. We used a standardized set of six questions concerning the aspects of ego disorders and graded the severity of symptoms using a 6-point Likert scale ranging from 0 = not present to 5 = very strong, so the sum score could range between 0 and 30 points. The resulting AMDP ego-disorder score showed a positive correlation with feelings of external influence (r_S_ = 0.403, *p* = 0.004, see Table 2). In patients with psychotic disorders, two further clinical scores were used to assess psychotic symptoms (PANSS = Positive and Negative Syndrome Scale^17^; SAPS = Scale for the Assessment of Positive Symptoms^18^). We found a significant correlation of average feelings of external influence across all conditions with the PANSS positive score (r_S_ = 0.409, *p* = 0.033) as well as with the SAPS total score value (r_S_ = 404, *p* = 0.035, see Table 2).

**Table 2:** Correlations of the mean intensity of external influence with psychopathological measures of psychotic symptom severity.

Score	Mean estimation of external influence
AMDP ego disorder score	r_S_ = 0.403 / **p = 0.004,** *n* = 43
PANSS positive score	r_S_ = 0.409 / **p = 0.033, ** *n* = 21
PANSS negative score	r_S_ = − 0.025 / *p* = 0.457, *n* = 21
PANSS general score	r_S_ = − 0.025 / *p* = 0.457, *n* = 21
SAPS total score	r_S_ = 0.404 / **p = 0.035, ** *n* = 21

AMDP = Arbeitsgemeinschaft für Methodik und Dokumentation in der Psychiatrie; PANSS = Positive and Negative Syndrome Scale; SAPS = Scale for the Assessment of Positive Symptoms. Given are Spearman’s Rho correlation coefficient (r_S_) and p values (p values < 0.05 are considered significant and printed in bold).

### Association of feelings of external influence with response latency

**Fig. 3:**
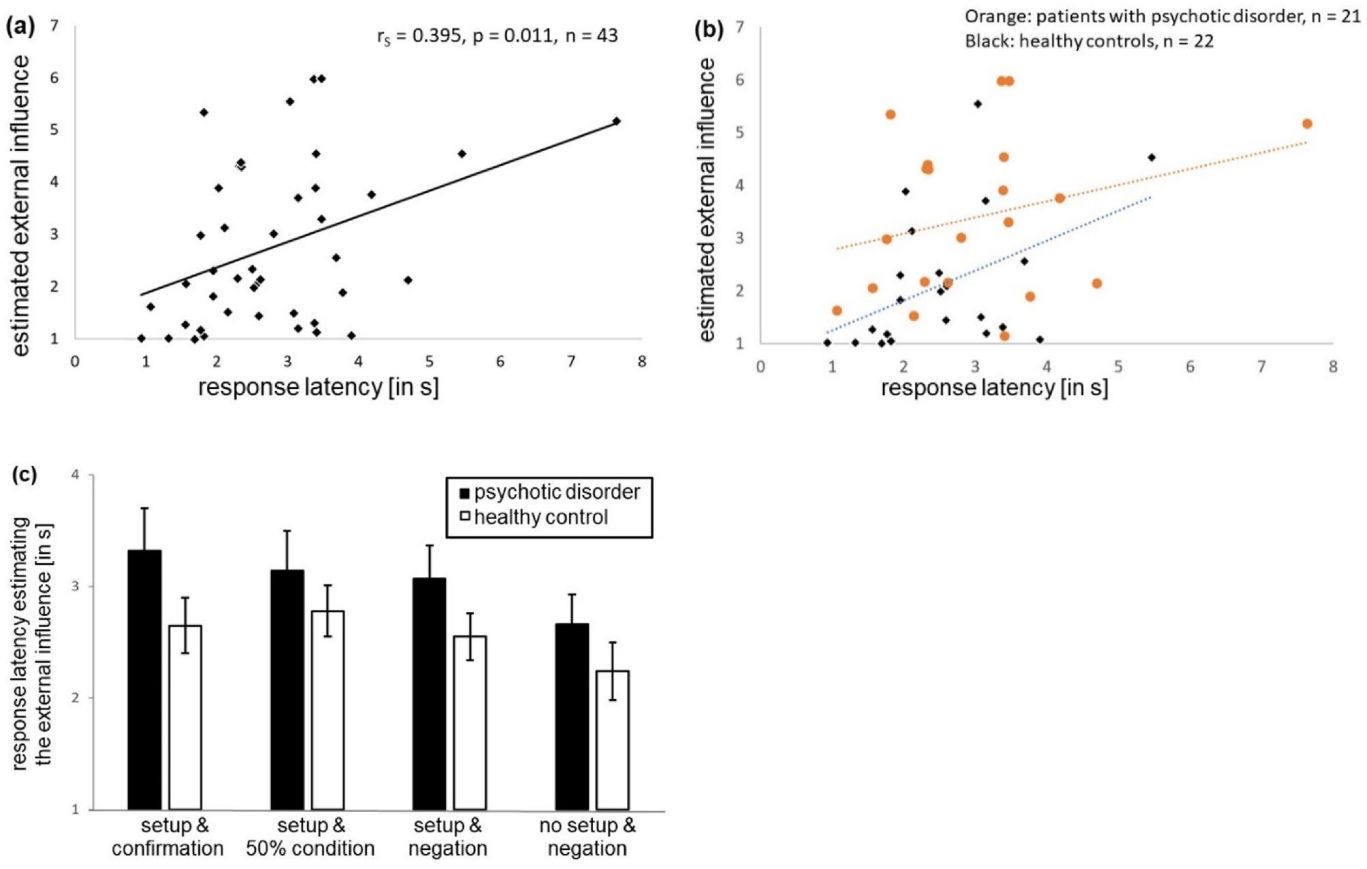
Estimation of external influence and response latency. (**a**) The average external influence rating per participant correlated significantly with the corresponding average response latency for estimating this influence. (**b**) In a subgroup analysis we confirmed the correlation in the group of healthy controls (black dots and blue punctuated line, *n* = 22), whereas in the group of patients with psychotic disorders (orange dots and orange punctuated line, *n* = 21) the significance of the correlation is absent. (**c**) Response latencies estimating the external influence differed between the different conditions “setup & confirmation”, “setup & 50% condition”, “setup & negation”, and “no setup & negation”. Black bars indicate patients with psychotic disorders, white bars healthy controls. Mean values and standard error of the means are depicted. s = seconds.

The response latencies when answering the question about the estimated external influence (mean value across all conditions) correlated positively with the estimated intensity of external influence across both groups (r_S_ = 0.395, *p* = 0.011, *n* = 43, see Fig. 3a), i.e., longer response latencies were associated with a higher intensity of felt external influence. Separated analysis showed a positive correlation for healthy controls (r_S_ = 0.450, *p* = 0.036, *n* = 22) but not for patients (r_S_ = 0.283, *p* = 0.213, Fig. 3b). However, the two correlation coefficients did not significantly differ between the groups (z = 0.5891, *p* = 0.556). The average response latencies did not differ between patients with psychotic disorder (3.05 ± 0.30 s) and healthy controls (2.55 ± 0.22 s) (F(1, 33) = 1.083, *p* = 0.306, partial ɳ_p_^2^ = 0.032), and no interaction effect showed significant differences (see Fig. 3c).

The Supplementary Material includes additional analysis on the intensity and emotional valence of the instructed imaginations as well as associations with specific object categories, i.e. whether a general object or a specific object with a more positive or negative connotation was imagined.

## Discussion

The current pilot study aimed to replicate and extend the results of our previous study with healthy subjects^12^ now in a clinical sample of patients with psychotic disorders as compared to healthy controls. To this end, 21 patients and 22 healthy controls participated in an imaginary task while feelings of external influence were induced using a physical setup (tDCS/hand touch vs. no setup) in combination with specific verbal information (external influence will be attempted vs. external influence will not be attempted). As intended by the experimental paradigm, we were able to show that the estimation of external influence can be modulated by the physical experimental setup as well as by the information given to the participants. Both modulating factors lead to an elevated feeling of external influence. The two different experimental setups (hand touch vs. tDCS) did not differ in their potential to modulate the feeling of external influence. As hypothesized, patients exhibited increased feelings of external influence in comparison to healthy controls across all experimental conditions.

These findings are well in line with previous paradigms investigating feelings of external influence or related constructs. For example, Klock and colleagues used a sham TMS device to manipulate feelings of authorship of imaginations^19^. As in our study, their results showed that the sham TMS device influenced the attribution of authorship depending on the information given to the participants. Furthermore, Olson and colleagues induced feelings of mind-influencing using a mock MRI neuroimaging scanner^20^. Participants reported higher involuntariness of thoughts during a mind-influencing task in comparison to a mind-reading task. In line with our results, the response latencies for decisions were longer in the mind-influencing task in comparison to the mind-reading task. As in our study, an external setting pretending to influence the thought led to a higher intensity of feelings of external influence. In another study by Walsh et al., hypnotic techniques were applied to healthy participants^21^. In this study, participants’ ratings of control, ownership and awareness of thoughts were reduced during an attempted influence in comparison to a control condition. The authors also included a “simulation condition” in which the participants should simulate that an engineer would influence them, while indeed he did not. No significant change in participants’ ratings was detected during the “simulation condition” relative to the voluntary condition^21^. These results also corroborate the concept in which reduction of thought ownership and increasing feelings of external influence can be induced by physical features of the experimental setup accompanied by explicit verbal information that influence will be attempted.

Interestingly, the results of this pilot study point to a differential contribution of the modulating factors “impact of information” and “impact of setup” on the estimation of felt external influence between patients with psychotic disorder and healthy controls. In healthy controls, the “impact of information” outweighed the “impact of setup”, whereas in patients with psychotic disorder the “impact of setup” significantly excelled the “impact of information”. This result represents a new and original finding as the above cited approaches and no other report found in our literature search did aim to disentangle the relative impact of these different components of intervention. Patients mainly seem to stick to the perceived physical aspects of the experimental setting, whereas subjects of the control group tend to orient their estimations of external influence more strongly on verbal information provided by the experimenter than on the presence of the physical setup. One possible explanation for this finding might be that patients with psychotic disorders show less trust in the information given by the experimenters but relied more on the physical environment compared to healthy controls. In line with this assumption, patients with psychotic disorders were observed to exhibit reduced baseline trust towards unfamiliar persons in interactive trust games, accompanied by a lack of adjustment in response to explicit verbal information about the trustworthiness of their interaction partner^22,23^. Moreover, a recent systematic review and meta-analysis confirmed a general reduction in trust towards others in patients with psychosis^24^. On the other hand, there is experimental evidence that belief formation may rely more strongly on initial perceptual impressions of the physical environment and that further information processing in patients with psychotic disorders is characterized by failures in perceptual updating^25^. For example, using a simple visual perception task that involved changes of dot motion direction, it was observed that the severity of psychotic symptoms is related to the inability to update beliefs based on new perceptual evidence^25^. Regarding auditory perception, it has been shown that auditory perceptual experience in patients with psychosis relied more on prior beliefs generated through presentation of written phonemes prior to presentation of ambiguous speech sounds as compared to healthy participants^26^. The stronger impact of prior beliefs and initial perceptual impressions on formation of perceptions and beliefs accompanied by impaired integration of subsequent inconsistent or even contradictory information is assumed to contribute to the development of delusional beliefs as well as hallucinations in patients with psychotic disorders^25,26^.

The responses obtained during the “setup & 50% condition” corroborate the successful blinding of the participants concerning the attempt of influence under this condition as well as the absence of any direct causal influence of actual intervention attempts on imaginations (low amplitude tDCS vs. sham-stimulation and hand touch with attempt to influence vs. hand touch without attempt to influence), since the correct detection rate of trials with attempted influence did not differ from chance level. Furthermore, the observation that trials with assumed influence were associated with a higher intensity of feelings of external influence confirmed the functioning of the paradigm.

The results of the healthy control group in the current study replicate previous findings in an almost identical approach with 60 healthy individuals^12^. In both studies, feelings of external influence were successfully induced by the physical setup as well as the verbal information component of the intervention. In the previous study, three different experimental setups (tDCS, eye contact and hand touch) successfully induced feelings of external influence without significant differences between these three setups. In accordance with the previous cohort of healthy individuals, the impact of information outweighed the impact of setup concerning the estimation of external influence. Regarding the “setup & 50% condition”, the previous study also provided evidence for successful blinding of the participants as well as relevant modulation of feelings of external influence associated with participants’ assumptions of influence attempts. A positive correlation between the estimated external influence and the response latency during the estimation process was also consistent in both cohorts.

In our study we apply a new experimental paradigm and ask for a rating of feelings of external influence during different experimental conditions. Additionally, established questionnaires assessing different aspects of proneness to delusional experience, absorption, attributional style, and severity of psychotic symptoms were assessed. We started with our experimental paradigm, so that participants were able to work through this without any former influence. We awaited that the following questionnaires would not be influenced by the preceding experimental paradigm, but we cannot exclude this possibility completely.

As a further limitation the small sample size of the current pilot study has to be mentioned. In a previous study with healthy controls 60 individuals were tested and small to medium effect sizes for the modulation of feelings of external influence could be revealed. The current study replicated the main findings of the previous study in healthy participants and demonstrated the feasibility of the experimental design for patients with psychotic disorders. Regarding the observed differential effects in patients and healthy controls, however, further research is warranted to confirm the findings.

If the results are confirmed, the current paradigm might have the potential to support diagnostics concerning delusions of influence and their modulation. To this aim, more research, including further correlations with diagnostic practices and specificity is needed. Already now the paradigm could be used for in-depth investigation of experienced external influence under the different experimental conditions in individuals with psychotic disorders. The paradigm can serve as a practical experienceable basis to discuss the variability and modulation of the feeling of external influence. Due to the possibility of experimental-based variation of the estimation of external influence and due to the well-defined experimental character of the experience, it may facilitate the communication and the critical questioning of these beliefs. The paradigm could therefore add to therapeutic interventions, thus expanding the applications of metacognitive training and contributing to further preventing the development of delusions.

## Methods

### Participants

We included 43 participants in the current study. The experiments were performed at the Department of Psychiatry and Psychotherapy, University Hospital Tübingen. Inclusion criteria comprised an age between 18 and 65 years and sufficient knowledge of the German language and normal or corrected to normal hearing and vision. Exclusion criteria were mental disability, known structural brain abnormalities or neurological disease. The healthy participants were recruited via email announcements, psychiatric disorders were excluded using the M.I.N.I^27^. None of the healthy participants took psychopharmacological medication. Patients with psychotic disorder were recruited from the Department of Psychiatry and Psychotherapy of the University Hospital Tübingen. The diagnoses (F20, F25) were confirmed according to the ICD-10 diagnostic criteria^28^. We included patients with diagnosis of schizophrenia (*n* = 16) and schizoaffective disorder (*n* = 5). All patients took psychopharmacological medication. Symptoms of psychotic disorder were assessed using the Positive and Negative Syndrome Scale (PANSS)^17^ and the Scale for the Assessment of Positive Symptoms (SAPS)^18^. For all participants, we additionally performed a structured psychopathological examination on the basis of the AMDP (Arbeitsgemeinschaft für Methodik und Dokumentation in der Psychiatrie) manual to quantify symptoms of ego disorders^14^. Specifically, six questions concerning different aspects of ego disorders described in the AMDP manual (derealization, depersonalization, thought withdrawal, thought insertion, thought broadcasting, other alienation symptoms). Participants were asked to rate the severity of each of these symptoms on a 6-point Likert scale from 0 = not present to 5 = very strong. A sum score was calculated as a measure of ego disorder symptoms, with AMDP ego disorder score reaching from 0 to 30 points. Additionally, all participants filled out questionnaires regarding estimated crystallized intelligence (measured with Multiple-choice Vocabulary Intelligence Test, MWT-B^13^), for attributional style (Attributionsstilfragebogen für Erwachsene, ASF-E^15^), proneness for delusional experience (Peters et al. Delusional Inventory, PDI^16^), , and absorption (Tellegen Absorption Scale, TAS^29,30^).

### Ethics statement

The study was conducted in accordance with the ethical principles of the Declaration of Helsinki (Code of Ethics of the World Medical Association) and was approved by the Ethics Committee of the Faculty of Medicine of the Eberhard Karls University and the University Hospital Tübingen (ethical approval number 662/2017BO2). Written informed consent was obtained from all participants prior to inclusion in the study. They received a small financial compensation for their participation (10 Euro per hour). Following the experiment, the participants were informed that no direct influence of the intervention on their imagination is to be expected, due to low amplitude of the current in tDCS and an absence of evidence for any direct influence on imaginations during hand touch; another written consent for further usage of the data was obtained (post-interventional informed consent).

### Stimulus material, task, and procedure

We used a recently established paradigm to assess the feeling of external influence during instructed imaginations^12^, which was slightly adapted. The experiments took place in a bureau with office furniture, which was otherwise empty. In the middle of the room stood a round table with a computer keyboard and screen on it and two chairs either opposite of each other or side by side, respectively. The investigator explained the experimental procedures, assembled and disassembled the experimental devices and attempted to influence the imagination of the participant during the hand touch condition (see below). First, the inclusion and exclusion criteria were checked, and the participants were informed verbally and in writing. If the participants gave their written informed consent, the investigation started with assessment of basic information (see also Fig. 1 upper row, left side, “Preparation”). The main experiment (see also Fig. 1 lower row) was presented via the software program Presentation (Neurobehavioral Systems Inc., Berkeley, CA, USA) on the computer. The explanation of the procedure was given written on the screen with additional explanations by the investigator if necessary. The participants were informed that they should imagine different objects, e.g. a balloon, for 10 s. The beginning and the end of the imagination period was signalled by a tone. Deviating from the previous study protocol, the participants were asked to close their eyes during the imagination. After each imagination, the participants were asked to open their eyes again. These instructions were given acoustically by a computer-generated female voice (MWS Reader + voice “Gudrun”, Digital River GmbH, Cologne, Germany). After the opening of eyes, the following questions presented on the computer screen: (1) “How intensively did you experience your imagination?”, (2) “How much was your imagination influenced by the intervention?”, (3) “Did your imagination trigger a pleasant or unpleasant feeling?”. In the “setup & 50% condition” (see below) a fourth question was posed: “Was there an attempt of influencing?”. For questions 1–3 participants got a 9-point Likert-scale ranging from 1 = very low/unpleasant to 9 = very high/pleasant. The participants gave their answers using the number keys (above the letter keys) on the keyboard of the computer. For the fourth question, the answers “Yes” and “No” were possible. For this purpose, the arrow keys (to the left and to the right) were labelled with “Yes” and “No”.

To manipulate the sensation of external influence, we introduced an intervention composed of two elements: an experimental setup randomized between participants and explicit verbal information given to participants, the order of the presentation randomized within subjects. As between-subject experimental setups we implemented (1) a Low-amplitude transcranial direct current stimulation (tDCS) intervention and (2) Skin-to-skin hand touch. Details concerning the setup can be found in the Supplemental Material. On a within-subject level, participants were provided with explicit verbal information regarding whether an attempt to influence them was made during the current trial with four different conditions: “setup & confirmation”: The setup was applied (either tDCS or hand touch), and participants were informed that an influence attempt was made; “setup & negation”: The setup was applied, but participants were informed that no influence attempt was made; “no setup & negation”: No external intervention setup was applied and participants were informed that no influence attempt was made. “setup & 50% condition”: The setup was applied (either tDCS or hand touch), and participants were informed that an influence would be attempted in 50% of trials, without knowing which ones. For further analyses, we calculated the “impact of information”, which represents the difference between the estimation of external influence in the condition “setup & confirmation” minus “setup & negation”, and the “impact of setup” as the difference of the estimated feelings of external influence between the conditions “setup & negation” and “no setup & negation”, which differ in the presence and absence of the setup, respectively. For each intervention, 60 trials were conducted, with conditions “setup & confirmation”, “setup & negation”, “no-setup and negation” comprising 12 trials each and condition “setup & 50%” involving 24 trials, equally split between influence attempt and no influence attempt in 12 trials each. Furthermore, we manipulated the specificity and emotional valence of the objects for imagination, categorizing them into 20 “general” objects (e.g., animal), 20 “specific positive” objects (e.g., rabbit), and 20 “specific negative” objects (e.g., spider). The 60 used object names can be found in Supplementary Table 1. All participants experienced both physical setups, typically in two separate sessions within a week, each lasting approximately one hour. The order of physical setups was randomized. After completion of the second session, in the last part of the study, the participants were asked to complete questionnaires. At the end, they were informed that no direct influence of the intervention on the imagination is to be expected. For this post-interventional information another written consent for further usage of the data was obtained (see also Fig. 1 upper row, right side, “Final assessment”).

### Statistical analysis plan

Data were analysed using SPSS version 28.0.0.0 (IBM Corporation, Armonk, NY, USA). For all analyses, p values < 0.05 were considered statistically significant. Normal distribution was tested using the Shapiro-Wilk-Test. The sociodemographic data, the answers of the questionnaires and the estimations of external influence, intensity and emotional valence of the imaginations were non-normally distributed. We performed Pearson-Chi-Quadrat tests for analyses of nominal values (sociodemographic data) and Fischer’s exact tests in cases with expected cell frequencies below 5. For metric variables of the sociodemographic and psychometric data, we performed Mann-Whitney U-tests. We applied repeated measures ANOVA, as this test is quite robust against biases for non-normally distributed data. Nevertheless, we added post-hoc testing for non-normally distributed data (Friedman test for repeated measures, Wilcoxon signed rank test for two dependent variables, Mann-Whitney U-test for two independent samples) to corroborate our results. Effect sizes of median values were calculated using r (r = |Z/$\surd n$|)^31^. Values of *r* < 0.3 indicate a small effect, between 0.3 and 0.5 an intermediate effect, and r values > 0.5 a strong effect. To account for multiple testing, p-values were corrected using the Benjamini-Hochberg procedure (FDR, false discovery rate). The accuracy rate of correct identification of attempted and not attempted trials in the “setup & 50% condition” was compared to chance level using a two-sided one sample t-test with a hypothesized value of 0.5. Comparison of groups was conducted using the Mann-Whitney U-test. Correlational analyses were performed using Spearman’s correlation coefficient for non-normally distributed data. Two-sided testing was applied if not stated otherwise. The comparison of the correlation coefficients between the two groups was performed using Fisher’s z transformation^32^.

## Electronic supplementary material

Below is the link to the electronic supplementary material.

Supplementary Material 1

## Data Availability

The datasets generated and analyzed during the current study are available from the corresponding author on reasonable request.
