# Supplementary material for: Increased feelings of external influence during instructed imaginations in patients with psychotic disorder
Source: Sci Rep. 2025 Jul 22;15:26669. doi: 10.1038/s41598-025-10439-7 (PMC12284240; doi:10.1038/s41598-025-10439-7)
Supplement: Supplementary file 1 — Supplementary Material 1 [file 41598_2025_10439_MOESM1_ESM.docx]

**Increased feelings of external influence during instructed imaginations in patients with psychotic disorder**

Kathrin N. Eckstein^1,2^, David Rosenbaum^1^, Anna Camera^1,3^, Lisa Röhrig^1,4^, Matthias L. Herrmann^1,5^, Dirk Wildgruber^1*^

^1^Department of Psychiatry and Psychotherapy, Tübingen Center for Mental Health (TüCMH), University of Tübingen, Tübingen, Germany

^2^Clinic for General Psychiatry and Psychotherapy, Zentrum für Psychiatrie (ZfP), Klinikum am Weissenhof, Weinsberg, Germany

^3^Translational Research Office, Medical Science Division, University of Oxford, Oxford, United Kingdom

^4^Center of Neurology, Division of Neuropsychology, Hertie-Institute for Clinical Brain Research, University of Tübingen, Tübingen, Germany

^5^Department of Neurology and Neuroscience, Medical Center, University of Freiburg, Freiburg, Germany

**Supplementary Material**

# Supplementary Details on Material and Methods

## Details concerning experimental setup and procedure

Low amplitude transcranial direct current stimulation (tDCS) using a tDCS device (neuroConn DC STIMULATOR, neuroCare Group GmbH, Ilmenau, Germany). The anode was placed over F3 according to the 10-20-EEG positioning reference, the cathode was placed on the right upper arm. The skin was cleaned and 5x7 cm electrodes were attached using electrode gel (ten20 Conductive Neurodiagnostic Electrode Paste, Weaver and Company, Aurora, CO, USA). The resistance was set below 10 kOhm. The amplitude was set to 0.4 mA, which is assumed to be below behavioural efficacy (Antal et al., 2017). Verum stimulation was applied during the 10 seconds of imagination, with additional 5 seconds for fade-in and fade-out, respectively. In an analogous manner, sham stimulation was applied with fading in and out during the fade-in and fade-out timeslot and no stimulation during the imagination period. Skin-to-skin hand touch of the dry and warm (between 30 and 34 °C measured with a surface temperature thermometer) palm of the hand of the investigator with the self-weight of the hand touching the back of the participant’s hand (during the 10 seconds of imagination). The staff member sat beside the participant and looked sideways past the participant. Two female students, who were previously unknown to the participants, led the experimental procedures and acted as influencers, as they attempted to influence the imagination in the hand touch condition.

## Details concerning objects of instructed imaginations

The participants were asked to imagine 60 different objects. The objects were grouped into three categories: general objects, specific objects with a more positive connotation and specific objects with a more negative connotation. The order was pseudorandomized, so the same category was never presented twice in a row and the three categories were balanced across the different experimental conditions.

| **General objects**  **(general)** | **Specific objects with a more positive connotation**  **(specific positive)** | **Specific objects with a more negative connotation**  **(specific negative)** |
| --- | --- | --- |
| Tier (Animal) | Hase (Rabbit) | Spinne (Spider) |
| Pflanze (Plant) | Rose (Rose) | Distel (Thistle) |
| Gebäude (Building) | Palast (Palace) | Ruine (Ruin) |
| Werkzeug (Tool) | Hammer (Hammer) | Zange (Tongs) |
| Flüssigkeit (Liquid) | Orangensaft (Orange juice) | Hustensaft (Cough syrup) |
| Möbel (Furniture) | Sessel (Armchair) | Hocker (Stool) |
| Gemüse (Vegetables) | Paprika ((Sweet) pepper) | Rosenkohl (Brussel sprouts) |
| Frucht (Fruit) | Erdbeere (Strawberry) | Zitrone (Lemon) |
| Kleidungsstück (Piece of clothing) | Handschuh (Glove) | Socke (Sock) |
| Straße (Street) | Allee (Boulevard) | Gasse (Alleyway) |
| Elektrogerät (Electrical device) | Fernseher (Television) | Radio (Radio) |
| Geschäft (Shop) | Feinkostladen (Delicatessen store) | Bahnhofskiosk (Train station kiosk) |
| Backzutat (Baking ingredient) | Zucker (Sugar) | Mehl (Flour) |
| Behälter (Container) | Handtasche (Handbag) | Plastiktüte (Plastic bag) |
| Heißgetränk (Hot beverage) | Kakao (Hot chocolate) | Ingwertee (Ginger tea) |
| Kosmektikartikel (Cosmetic product) | Parfüm (Perfume) | Pinzette (Tweezers) |
| Fahrzeug (Vehicle) | Limousine (Sedan) | LKW (Semi-trailer) |
| Schiff (Ship) | Segelboot (Sailing boat) | U-Boot (Submarine) |
| Musikinstrument (Musical instrument) | Gitarre (Guitar) | Flöte (Flute) |
| Gewürz (Spice) | Vanilleschote (Vanilla bean) | Chilischote (Chili) |

# Supplementary Table 1. Objects for imagination task.

# Supplementary Results

## Effects of conditions on intensity and emotional valence of instructed imaginations

In addition to estimations of their feelings of external influence, participants were asked to rate the intensity and emotional valence of the instructed imaginations during the different task conditions.

The intensity of instructed imaginations was rated 6.43 ± 0.16 during “setup & confirmation”, 6.35 ± 0.17 during “setup & 50% condition”, 6.44 ± 0.14 during “setup & negation”, and 6.48 ± 0.14 during “no setup & negation”. We did not find a difference in the intensity estimation between the different conditions (F(3, 99) = 0.576, p = 0.632, partial ɳ_p_^2^ = .017, Supplementary Figure 1a).

Patients with psychotic disorder rated the intensity on average 6.15 ± 0.21, healthy controls 6.69 ± 0.18. RM ANOVA reported a significant difference detectable between the two groups, with healthy controls showing higher imagination intensities than patients with psychotic disorder (F(1, 33) = 4.550, p = 0.004, partial ɳ_p_^2^ = .121), but this result was not confirmed by nonparametric post-hoc testing (Z = -1.762, p = 0.079, r = 0.269), therefore no significant difference can be stated between groups. The interaction condition*group was also not significant (F(3, 99) = 2.361, p = 0.076, partial ɳ_p_^2^ = .067, Supplementary Figure 1b)

The emotional valence amounted to 5.86 ± 0.09 during “setup & confirmation”, to 5.83 ± 0.11 during “setup & 50% condition”, to 5.95 ± 0.11 during “setup & negation”, and to 5.91 ± 0.11 during “no setup & negation”. We did not detect a difference in the emotional valence estimation between the different conditions (F(3, 99) = 0.640, p = 0.591, partial ɳ_p_^2^ = .019, Supplementary Figure 1c). The mean emotional valence in patients with psychotic disorder was 5.82 ± 0.14, in healthy controls 5.95 ± 0.12. Concerning the emotional valence judgement the groups did not statistically significantly differ (F(1, 33) = 0.129, p = 0.772, partial ɳ_p_^2^ = .004). There was no interaction condition*group (F3, 99) = 1.682, p = 0.176, partial ɳ_p_^2^ = .048, Supplementary Figure 1d).

**
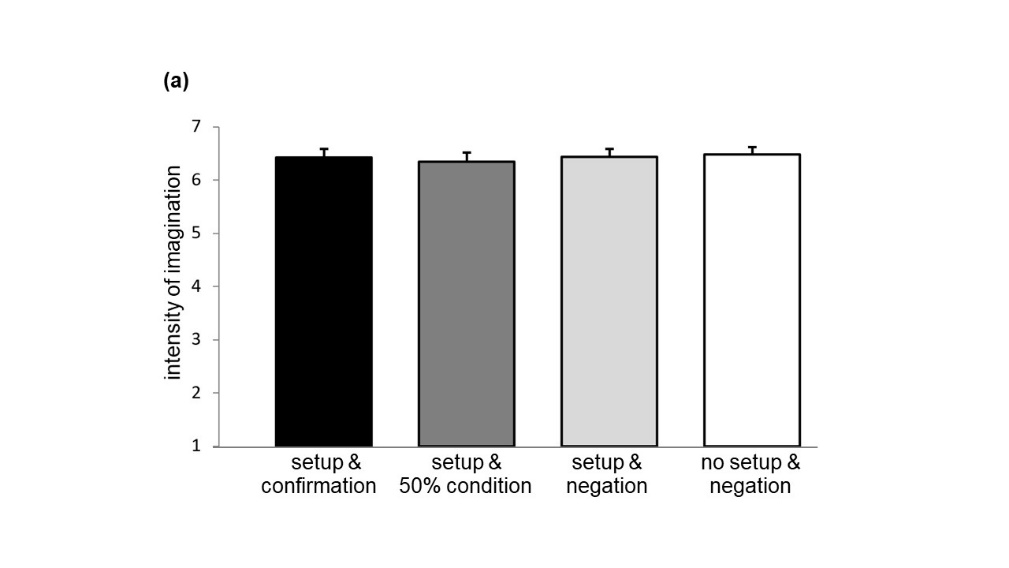

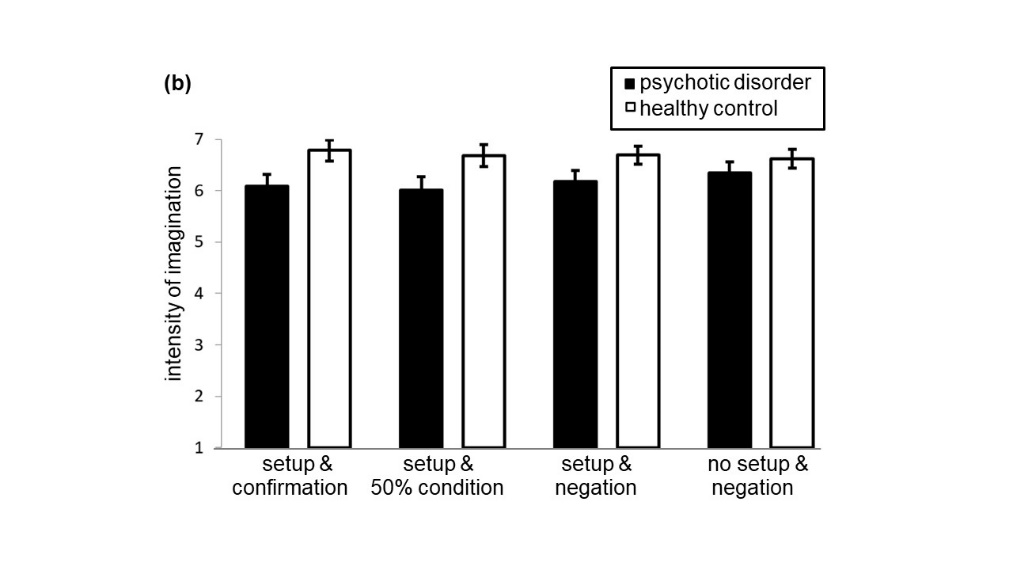

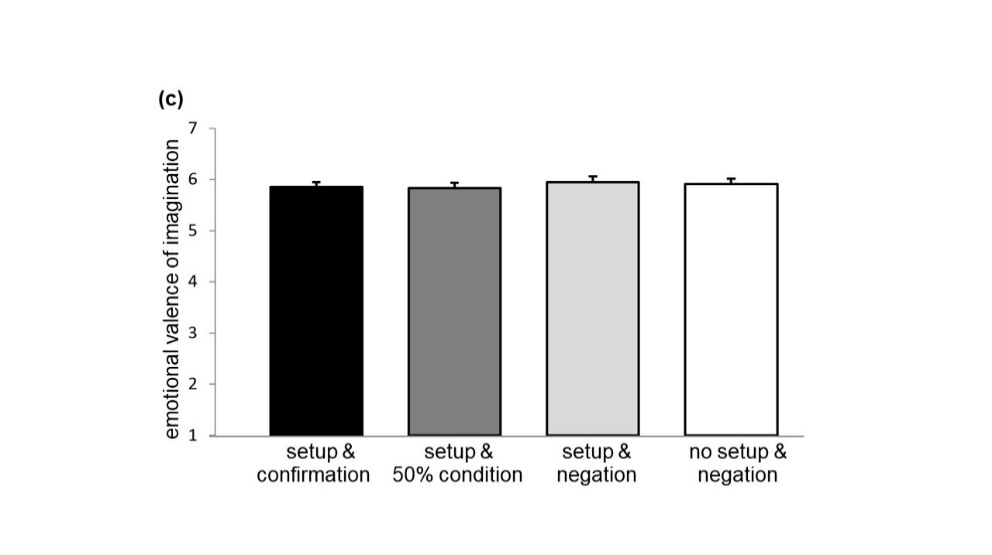

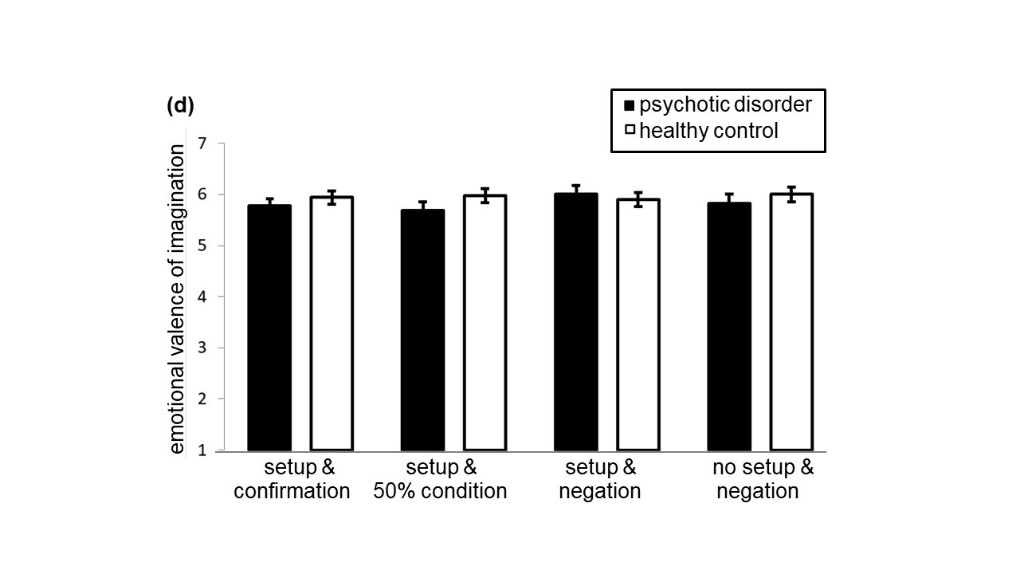
**

**Supplementary Figure 1. Estimation of intensity and emotional valence of instructed imaginations during different conditions and between the groups**

(a) Intensity of the imaginations and (c) emotional valence of the imagination during the four different conditions “setup & confirmation”, “setup & 50% condition”, “setup & negation”, “no setup & negation” based on the total participants’ sample (n = 43). In Figure (b) intensity of imagination and in figure (d) emotional valence of the imagination results are split into the two groups. Patients with psychotic disorder are depicted in black bars, healthy controls in white bars. Statistically significant differences were found neither between conditions nor between groups. Mean values and standard error of the means are depicted.

## Effects of object categories on emotional valence of instructed imaginations, feelings of external influence and intensity of imaginations

The objects presented audibly for imagination can be categorized into three groups (Supplementary Table 1). We presented 20 general objects (general), 20 objects with a more positive connotation (specific positive), and 20 objects with a more negative connotation (specific negative).

We asked the 43 participants to rate the emotional valence of their imagination after each trial. The emotional valence was on a 9-point Likert-scale ranging from 1 = very unpleasant to 9 = very pleasant rated on average 6.04 ± 0.10 for general objects, 6.14 ± 0.11 for specific positive, and 5.53 ± 0.09 for specific negative objects. The RM ANOVA analysis verified a difference in the emotional valence of the imaginations between the categories (F(1,617, 64.668) = 43.719, p < 0.001, partial ɳ_p_^2^ = .522). Nonparametric post-hoc tests confirmed differences between general and specific negative (Z = -5.181, p < 0.001, r = 0.790) and specific positive and specific negative objects (Z = -5.223, p < 0.001, r = 0.797, Supplementary Figure 2a). The groups, i.e. patients with psychotic disorder and healthy controls, did not statistically differ (F(1, 40, = 0.492, p = 0.487, partial ɳ_p_^2^ = .012, data not shown). The interaction category*group was not significant (F(1.617, 64.668) = 0.759, p = 0.447, partial ɳ_p_^2^ = .019, Supplementary Figure 2b).

The estimation of external influence ranged between 2.77 ± 0.23 for general objects, 2.78 ± 0.23 for specific positive, and 2.76 ± 0.23 for specific negative objects. Statistical analysis did not reveal a significant difference between the three different object categories (F(1.602, 62.496) = 0.121, p = 0.842, partial ɳ_p_^2^ = .003, Supplementary Figure 2c). As shown before estimation of external influence differed between the two groups (F(1, 39 = 6.783, p = 0.013, partial ɳ_p_^2^ = .148, data shown in the main manuscript Figure 1). The interaction category*group was not significant (F(1.589, 63.544) = 0.696, p = 0.471, partial ɳ_p_^2^ = .017, Supplementary Figure 2d).

The intensity of the imagination was estimated 6.47 ± 0.16 for general objects, 6.50 ± 0.14 for specific positive, and 6.36 ± 0.15 for specific negative objects. The intensity did not statistically differ between categories (F(1.415, 56.593) = 2.379, p = 0.118, partial ɳ_p_^2^ = .056, Supplementary Figure 2e). No difference between groups was observed (F(1, 40) = 1.480, p = 0.123, partial ɳ_p_^2^ = .058). The interaction category*group was not significant (F(1.415, 56.593) = 0.070, p = 0.872, partial ɳ_p_^2^ = .002, Supplementary Figure 2f).


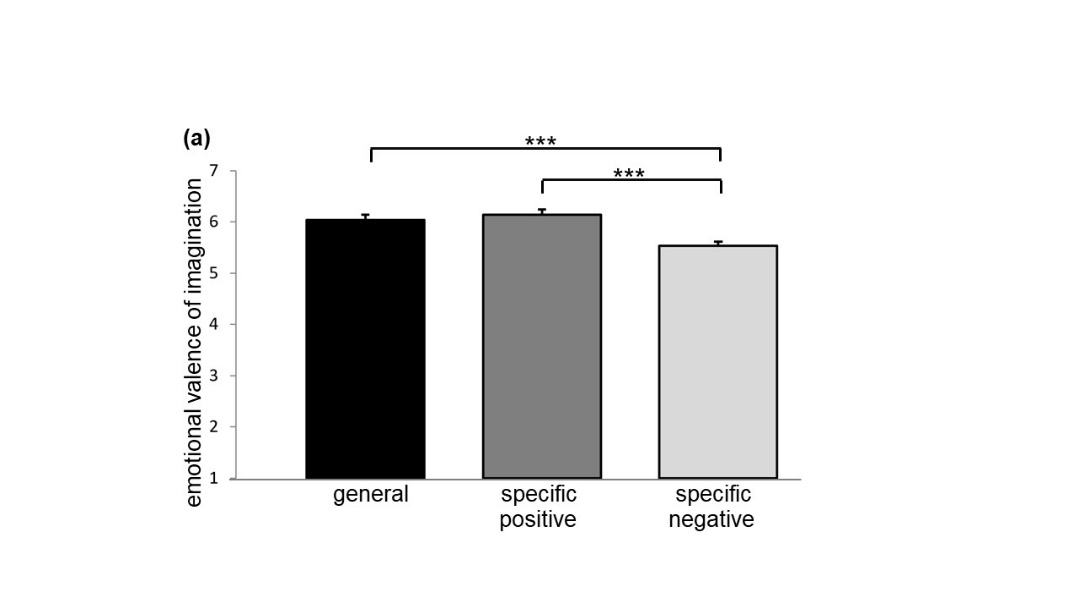

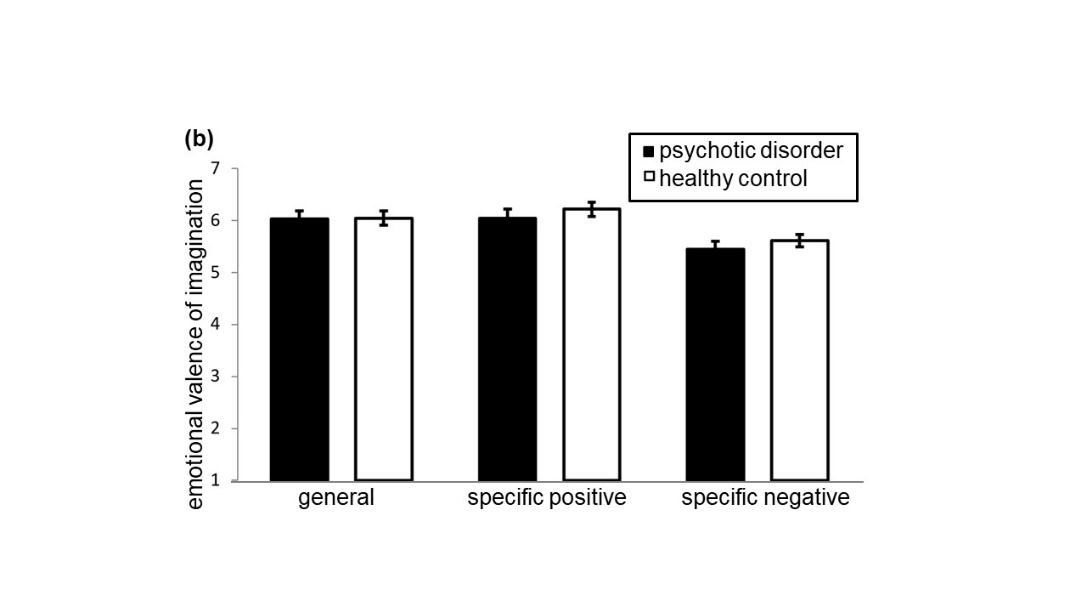

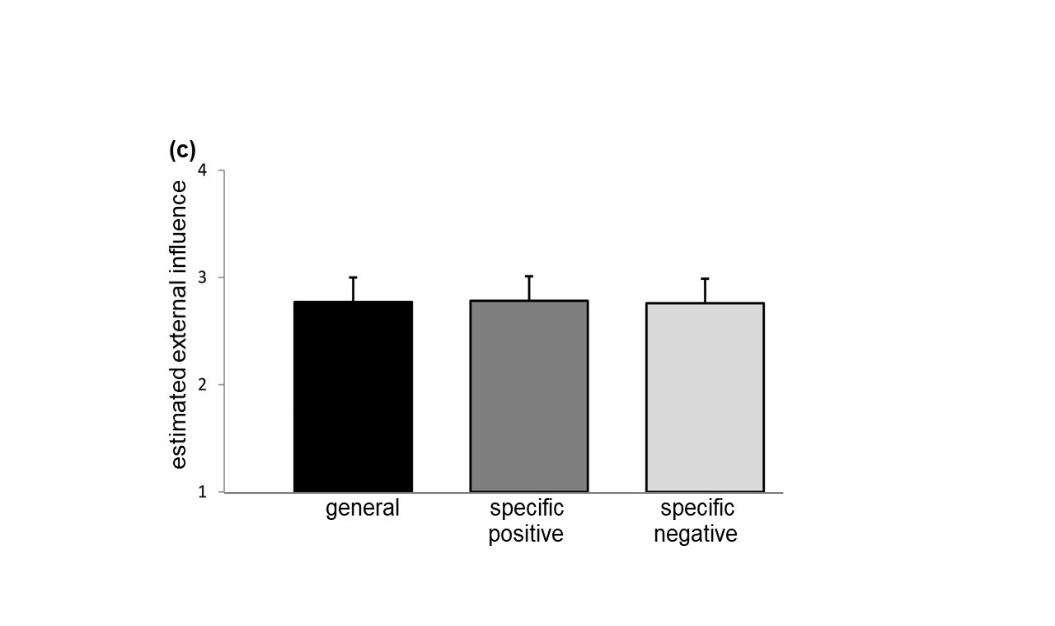

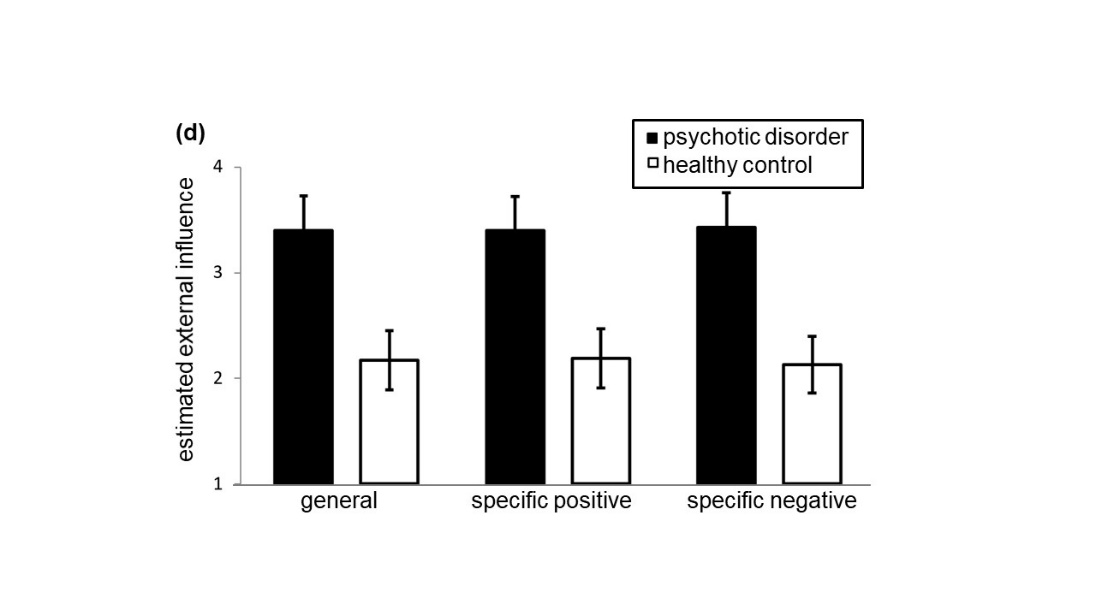

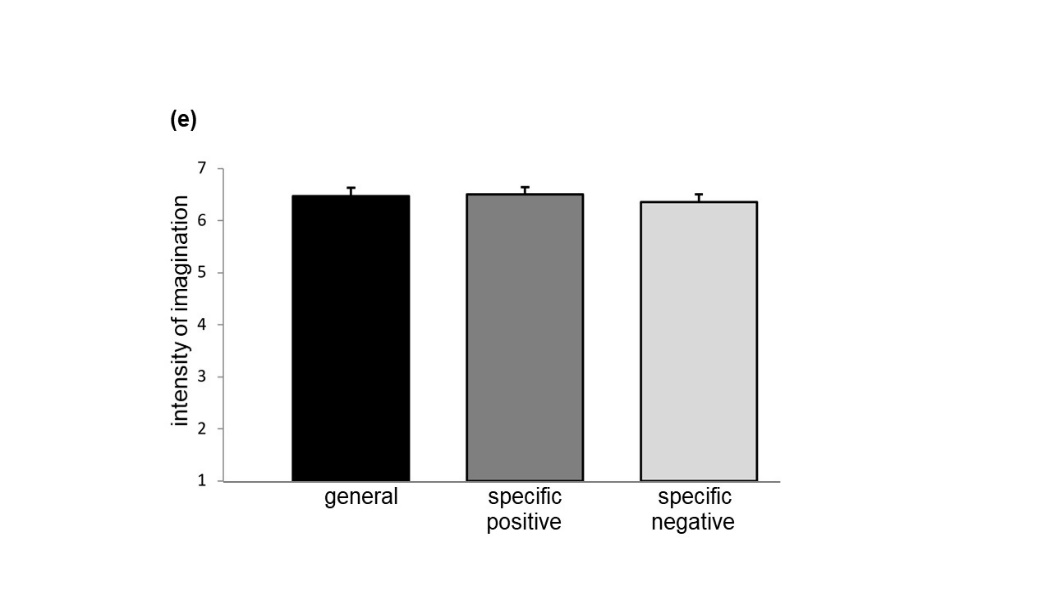

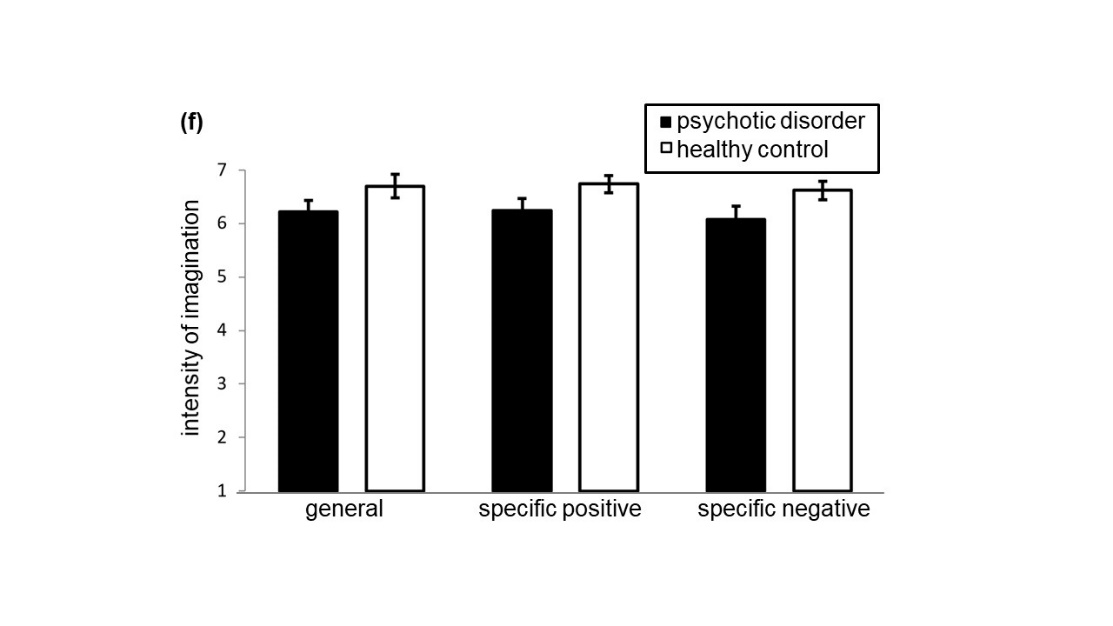


**Supplementary Figure 2. Emotional valence of imaginations, estimated external influence and intensity of imaginations for the different object categories (general, specific positive, specific negative).**

(a) Mean emotional valence, (c) estimated external influence, and (e) Intensity of the imagination of the total sample (n = 43) are analyzed concerning the different object categories: in black for general objects, dark grey for specific positive objects and light grey for specific negative objects. (b), (d) and (f) show emotional valence, estimated external influence and intensity for patients with psychotic disorder (black) and healthy controls (white) separately. Please consider the different scaling of the y-axis between a/e vs. c and b/f vs. d. Mean values and standard error of the means are depicted, *** p < 0.001.

# References

Antal, A et al. Low intensity transcranial electric stimulation: Safety, ethical, legal regulatory and application guidelines*. Clinical Neurophysiology* **128**, 1774-1809, doi:10.1016/j.clinph.2017.06.001 (2017).
